# Thermoplastic Disks Used for Commercial Orthodontic Aligners: Complete Physicochemical and Mechanical Characterization

**DOI:** 10.3390/ma13102386

**Published:** 2020-05-22

**Authors:** Valeria Daniele, Ludovico Macera, Giuliana Taglieri, Alessandra Di Giambattista, Giuseppe Spagnoli, Alessandra Massaria, Massimo Messori, Enrico Quagliarini, Gianluca Chiappini, Vincenzo Campanella, Stefano Mummolo, Enrico Marchetti, Giuseppe Marzo, Vincenzo Quinzi

**Affiliations:** 1 Department of Industrial and Information Engineering and Economics, University of L’Aquila, Piazzale Pontieri 1, Monteluco di Roio, 67100 L’Aquila, Italy; valeria.daniele@univaq.it (V.D.); giuliana.taglieri@univaq.it (G.T.); digiambaale@hotmail.it (A.D.G.); giuseppe.spagnoli@univaq.it (G.S.); 2 Department of Life, Health & Environmental Sciences, Postgraduate School of Orthodontics, University of L’Aquila, P.le Salvatore Tommasi 1, Ed. Delta 6, 67100 L’Aquila, Italy; massariaalessandra@gmail.com (A.M.); stefano.mummolo@cc.univaq.it (S.M.); enrico.marchetti@cc.univaq.it (E.M.); giuseppe.marzo@univaq.it (G.M.); vincenzo.quinzi@univaq.it (V.Q.); 3 Department of Engineering ‘Enzo Ferrari’, University of Modena and Reggio Emilia, Via P. Vivarelli 10, 41125 Modena, Italy; mmessori@unimore.it; 4 Department of Construction, Civil Engineering and Architecture, Polytechnic University of Marche, 60121 Ancona, Italy; e.quagliarini@staff.univpm.it; 5 Department of Industrial Engineering and Mathematical Sciences, Polytechnic University of Marche, via Brecce Bianche snc, 60131 Ancona, Italy; g.chiappini@univpm.it; 6 Department of Clinical Science and Translational Medicine, University of Rome “Tor Vergata”, Via Montpellier 1, 00133 Roma, Italy; vincenzo.campanella@uniroma2.it

**Keywords:** thermoplastic materials, invisible orthodontic appliances, physicochemical characterization, mechanical properties, colour change evaluations, water absorption behaviour

## Abstract

Invisible orthodontic aligners (IOAs) have been introduced in the orthodontic field as an innovative alternative for fixed brackets, in relation to their ability to be easily inserted/removed from the oral cavity without affecting the chewing ability and the aesthetic of the patients. The paper provides a complete physicochemical and mechanical characterization of thermoplastic materials in the form of disks used for commercial IOAs. A wide palette of specific techniques is considered, from tensile tests and dynamic-mechanical analysis, to X-Ray diffraction (XRD), differential scanning calorimetry (DSC), Fourier transformation infrared spectroscopy (FTIR-ATR) analyses and water absorption tests. The disks are investigated before and after immersion into staining beverages (red wine, coffee, nicotine and artificial saliva), in terms of colour variations, transparency, and microscopic surface modifications by means of colorimetry, UV-VIS absorbance and scanning electron microscopy (SEM). Among all the samples, polyurethane (PU) exhibited the highest crystallinity and the highest values of mechanical and thermal resistance, while the poly(ethylene terephthalate)-glycol (PETG) samples presented better transparency and less ability to absorb water. Moreover, red wine and coffee give noticeable colour variations after 14 days of immersion, together with a slight reduction of transparency.

## 1. Introduction

With an increase in the demand for adult orthodontics, the demand for invisible orthodontic appliances (IOAs) that can replace the commonly used metal brackets is also increasing [1]. Conventional orthodontic brackets frequently increase the risk of carious lesions and cause gingivitis and periodontitis because of surrounding plaque accumulation [2]. This results in impaired oral health in addition to poor aesthetics during orthodontic treatment [3]. To remedy this problem, invisible orthodontic aligners (IOAs) have been introduced as alternatives for fixed brackets and wires. Invisible orthodontic appliances can be easily inserted and removed and do not affect the chewing ability of the patient [4]. Tooth movement without the use of bands, brackets, or wires was described as early as 1945 by Kesling, who reported the use of a flexible tooth positioning appliance [5,6,7,8]. Subsequently, Sheridan and other researchers developed various types of invisible retainers. Align Technology, Inc. (Santa Clara, CA, USA) introduced the Invisalign system two decades ago, which further developed the principles of Kesling, Nahoum and other researchers using computer-aided design (CAD)/computer-aided manufacturing (CAM) technology combined with laboratory techniques that facilitated the fabrication of a series of dental devices with customized aesthetics that were removable and could move teeth from the beginning to the end [9]. Nevertheless, several thermoplastic materials are considered and the majority of current aligner manufacturers use modified polyethylene terephthalate glycol (PETG) [10,11,12], but comparative analyses that provide useful indications to make decisions based on scientific features are scarce or absent in literature. Considering the ever-growing attention in the dentistry field towards the use of invisible orthodontic aligners, further research on the thermoplastic materials used to manufacture aligners is necessary. 

For this task, the present paper aims to provide, for the first time, a complete physicochemical and mechanical characterization of the materials in the form of disks used for the fabrication of IOAs. In particular, the thermoplastic disks here considered are made from two commercial materials, poly(ethylene terephthalate)-glycol (PETG), an analogue/derivative to polyethylene terephthalate (PET), and polyurethane (PU). Among the PETG-based materials, three samples, coming from different brands, are considered in order to evaluate their differences, in terms of degree of crystallinity, optical properties—useful to identify the material with the best characteristics in terms of transparency—and mechanical features in terms of hardness and elastic modulus. Moreover, considering that the exposure of orthodontic materials to staining agents could cause unfavourable and unaesthetic colour changes [13,14,15,16,17], the present paper is also focused on analysing the colour stability of the PETG and PU-based materials, before and after exposure of staining solutions. Finally, the behaviour towards water absorption is investigated at different temperatures, given that the temperature variation plays a fundamental role in the diffusion of water molecules inside the polymeric structure and it is responsible for swelling and mechanical degradation phenomena in the material itself [18,19,20,21,22]. 

In this regard, the present work gives original and significant analyses in relation not only to the water absorption measurements but also to the solubility of the disks when exposed at high temperature but also at the typical temperature of oral cavity (37 °C). Finally, beside the scientific aspects related to the materials properties, this investigation also presents a useful resource for dentistry operators and manufacturing companies in evaluating and predicting the performance of these products, in addition to the information reported in technical sheets (which are not always readily available before purchasing the products). 

## 2. Materials and Methods

### 2.1. Chemicophysical Characterization of the As-Received Disks

The thermoplastic materials, employed for the realisation of the transparent orthodontic aligners, came from different brands and are characterized by several features in relation to their chemical composition, as reported in Table 1. 

Before starting the physicochemical characterization, each thermoplastic material available in the form of disk was divided into six equal segments (Figure S1) and each part was characterized by means of different techniques: X-Ray diffraction (XRD), differential scanning calorimetry (DSC), Fourier transformation infrared spectroscopy (FTIR-ATR), UV-visible spectrophotometry and scanning electron microscopy analyses (SEM). In particular, the XRD analysis, performed to establish the degree of crystallinity and the phase composition, was carried out by means of a PANalytical X’Pert PRO (PANalytical Inc., Almelo, Netherlands) diffractometer equipped with a Ni filter (CuKα radiation), in the 5–70° 2θ exploration range and considering a constant steps of 0.026° 2θ (time per step 400 s). XRD patterns were elaborated using a Profile Fit software (HighScorePlus software package, version 4.6a, PANalytical Inc., Almelo, The Netherlands), and crystalline phases were attributed by ICDD (ICDD, Philadelphia, PA, USA) and ICSD (FIZ Karlsruhe GmbH, Eggenstein-Leopoldshafen, Germany) reference databases. 

The thermal characterization analyses (DSC) were performed using a differential scanning calorimeter (Perkin Elmer DSC 8500, PerkinElmer, Waltham, MA, USA), heating the samples in the temperature range −70–240 °C at a rate of 10 °C min^−1^ under a dry nitrogen atmosphere. Then, from the second heating the glass transition temperature (Tg) was determined.

Regarding the Fourier transform infrared spectroscopy (FTIR-ATR), the measurements were performed by using a NexusTM 870 FT-IR spectrophotometer (Thermo Fisher Scientific, Waltham, MA, USA), over a range of 500–4000 cm^−1^ at a resolution of 2 cm^−1^. The ATR accessory was equipped with a monolithic diamond.

**Figure 1:**
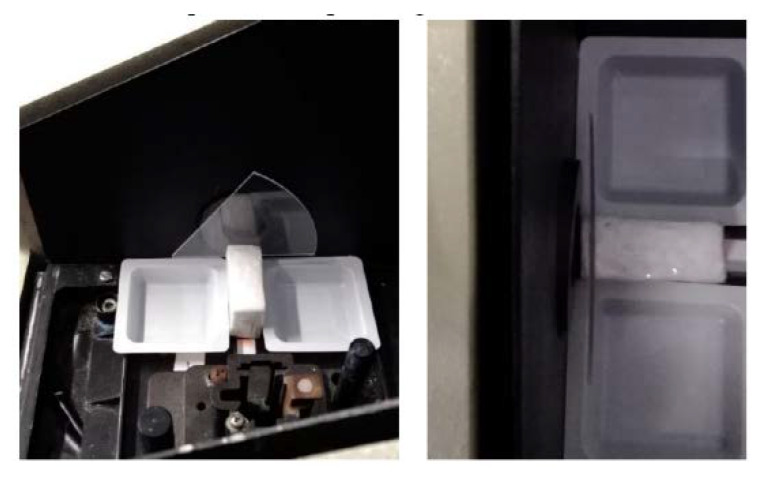
The ad hoc polystyrene support realised to correctly position the sample inside the UV-visible instrument.

In order to investigate the optical properties of each considered sample, useful to identify the material with the best characteristics in terms of transparency, the absorbance A was measured in the visible light (λ = 300–900 nm) by means of a Lambda 25 Perkin-Elmer ultraviolet/visible spectrophotometer (PerkinElmer, Waltham, MA, USA). The analysis was carried out in transmission, by positioning the disk on the ad hoc polystyrene support perpendicularly to the incident beam. In particular, the presence of the polystyrene support is crucial to collocate the disk in the same position during each analysis, in order to guarantee the transmission by the same area of the disk, so as to control the reproducibility of repeated analyses and to compare data before and after the immersion in the staining aging solutions (Figure 1). This is an important factor to be considered to achieve good and reproducible results, because a very little shift during the measurement corresponds to a great variation of the absorbance value.

### 2.2. Mechanical Characterization of the As-Received Disks

**Figure 2:**
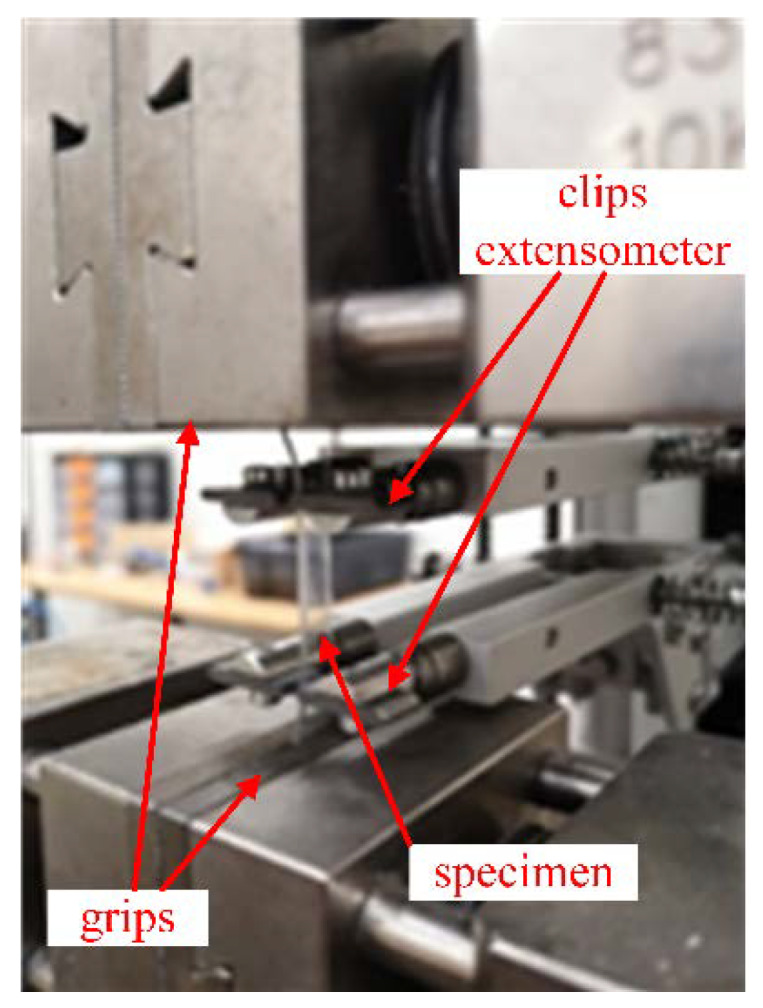
Representative tensile test on bone-shaped samples.

The tensile test and dynamic-mechanical analysis were performed to characterise the main mechanical properties of the as-received disks by following previous results from literature [11,19,21,26,27,28,29,30] and UNI EN ISO 527-2 [31]. The tensile test was completed using the same thickness as the manufacturer, and the specimens were bone-shaped (geometry 1BA from standard UNI EN ISO 527-2 [31]) and their gauge was 6 mm wide and 30 mm long. The quasi-static tensile tests were conducted by a standard electromechanical machine (model Zwick/Roell® Z050, Zwick Roell, Genova, Italy) imposing a crosshead speed of 5 mm/min [32,33]. The force was measured with a load cell of 5 kN and a high-resolution extensometer for precise measurement of the elongation within the gauge length was used (Figure 2). All tests were conducted at room temperature (about 20 °C).

Dynamic-mechanical analysis was performed by using a TA DMA Q800 analyser (TA Instruments, New Castle, DE, USA) in single cantilever configuration. Tests were carried out from −60 °C to 100 °C with a heating rate of 3 °C min^−1^, an oscillating frequency of 5 Hz and an applied strain of 0.2%. Due to the presence of frozen molecular orientation deriving from the manufacturing process, all the tested specimens exhibited a significant shrinkage above glass transition temperature (in the range 80–100 °C) which in turn caused the loss of a reliable instrumental signal. For this reason, the analysis was limited to temperatures lower than 100 °C in order to obtain information on the glassy state of the material. The tests were repeated twice on 5 mm × 40 mm specimens, using the same thicknesses as the manufacturer.

### 2.3. Immersion into Staining Beverages: Colour Change Evaluations.

As the colour stability of orthodontic materials can be unfavourable influenced by staining beverages, subsequently causing aesthetic changes related to the loss of transparency, the aim of this paper is to analyse and compare the colour stability of the materials, before and after the exposure of staining solutions. In particular, the considered samples were immersed into three staining solutions (red wine, coffee and nicotine) and into artificial saliva used to simulate intraoral aging. The staining solutions were prepared as follows: (1) undiluted red wine (San Crispino red wine, Cantine Ronco); (2) 3 g of coffee powder (Nescafè Classic instant coffee) were added in 100 mL of boiling distilled water; (3) the nicotine solution, properly filtered, was obtained by infusing cigarettes filters into distilled water; (4) 120 mL of Oral Balance artificial saliva (Biotène Oral Balance, GlaxoSmithKline Consumer Healthcare S.p.A.) was diluted in 480 mL of deionized water.

Four pieces of thermoplastic discs from each brand were randomly selected and divided into four groups according to the staining solutions. The samples were maintained in immersion up to 14 days in a water bath at T = 37 °C, and subsequently characterized in terms of FTIR-ATR, UV-VIS and colour change. In particular, the colour changes before and after the immersion were evaluated according to the Commission Internationale de l’Eclairage L*a*b* colour system (CIE L*a*b*) [16,34,35], where the parameter L* corresponds to the lightness (+ bright, − dark) while the a* and b* parameters indicates the colour scale from red (+) to green (−) and yellow (+) to blue (−), respectively [34,35]. The colour change values were determined by means of PCE Instrument colourimeter (PCE Deutschland GmbH, Meschede, Germany), before staining (t0) and after 7 and 14 days of immersion (t7 and t14, respectively). All the samples were washed in an ultrasonic bath for 5 min and then dried with paper before starting the measures. The colour measurements were then performed by maintaining physical contact between the vertical tip of the optical sensor and the sample surface. The total colour change (called ΔE*), representing the colour difference before and after staining, was finally calculated according to the equation [34,36]:

ΔE* = [(ΔL*)^2^ + (Δa*)^2^ + (Δb*)^2^]^1/2^ (1)

After the measurements, all the data were converted into the national bureau of standards (NBS) system by using the equation NBS = ΔE* × 0.92, in order to describe the levels of perceivable colour change under visual inspection [37,38]. In particular, according to the NBS system, the colour variations can be considered as perceivable if they are higher than 1.5, as reported in Table 2.

### 2.4. Water Absorption Test at Different Temperatures

Standard tests of water absorption were carried out, according to the International Organization for Standardization, on specimens of the thermoplastic materials [39,40]. In particular, for each manufacturer, three specimens were stored for 14 days at different temperatures (T), as follows: 20 °C ± 1° C, 37 °C ± 1 °C and 70 °C ± 1 °C respectively, in order to evaluate the influence of the temperature on the mechanical cohesion decrease. Then, both the water absorption (Wsp) and the water solubility (Wsl) were calculated by means of the following equations, in accordance with the corresponding ISO:

$$W_{sp}=\frac{m_{2}-m_{3}}{V}$$

$$W_{sl}=\frac{m_{1}-m_{3}}{V}$$

where *m*_1_ is the mass of the disks before the immersion in water, *m*_2_ is the mass during the immersion period up to 14 days and *m*_3_ is the mass after reconditioning and V is the volume of the considered aligner for this test. Two disks for each brand were considered, and the medium value of the obtained results was reported. All the data are expressed in μg/mm^3^. Finally, the as-received thermoforming materials, as well as those immersed both in water at different temperatures and in staining beverages, were observed by scanning electron microscopy analyses (SEM, Gemini SEM 500, Zeiss, Oberkochen, Germany), in order to investigate the surface modifications. For this task, the immersed pieces of thermoplastic discs from each brand were cut, chrome coated in a sputter coating unit, and observed at different magnifications.

## 3. Results and Discussion 

### 3.1. Chemicophysical and Mechanical Characterization of the Disks

**Figure 3:**
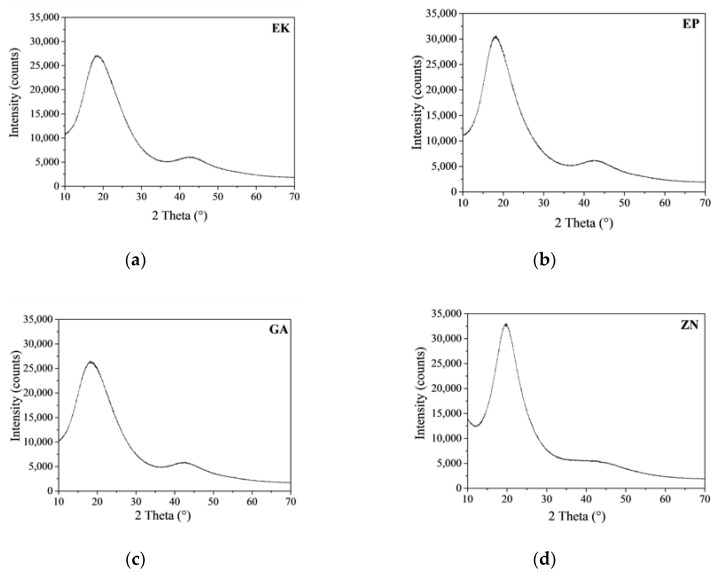
X-ray diffraction (XRD) spectra of the as-received thermoplastic materials: (**a**) EK; (**b**) EP; (**c**) GA; (**d**) ZN.

Figure 3 shows the X-ray diffraction patterns of the as-received thermoplastic materials of the different brands.

It is evident that EK, EP and GA samples have comparable XRD patterns, and this result confirms that they are made up of amorphous PET, the same base polymer also associable with PETG, by the comparison with the ICDD reference database (#00-060-1509) [41,42]. In fact, we observed the presence of two broad diffraction haloes that peaked at around 19° and 43° 2θ, respectively, and that were exactly related to the features of the amorphous PET phase. On the contrary, the ZN sample is composed of a different based-polymer, revealing an XRD pattern characterized by three broad haloes at around 10°, 20° and 43° 2θ, assigned to the scattering from soft polyurethane (PU) chains with regular interplanar spacing [43,44]. In Table 3 the measurement of the full width at half maximum (FWHM) of the Bragg peaks is reported, which allows one to determine the degree of crystallinity of the considered samples. The obtained results reveal that ZN disk presents the lowest FWHM value (corresponding to 6.313° 2θ) among all the samples; EK and GA disks show comparable FWHM values, while EP presents a lower FWHM value, resulting in the highest crystalline material among all the PETGs. 

The DSC curves, performed to determine the glass transition temperature (Tg), confirm the XRD results, revealing that all the samples are made by amorphous thermoplastics. Only one thermal phenomenon, corresponding to the Tg, can be recognised (Figure S2). In particular, EK, EP and GA samples are characterized by endothermic peaks having Tg values of 83 °C, 89 °C and 80 °C respectively, corresponding to the typical Tg of PETG polymers. In fact, as reported in literature, PETG is considered an amorphous thermoplastic of commercial PET, having a glass transition temperature of about 80 °C [22,45]. On the contrary, the glass transition temperature of the ZN sample is of about 96 °C, a value different both from the Tg values generally associated with PETG and PU based-polymers. This is probably attributed to the additives present into the polyurethane-based mixtures, as also discussed in a previous literature [46]. 

**Figure 4:**
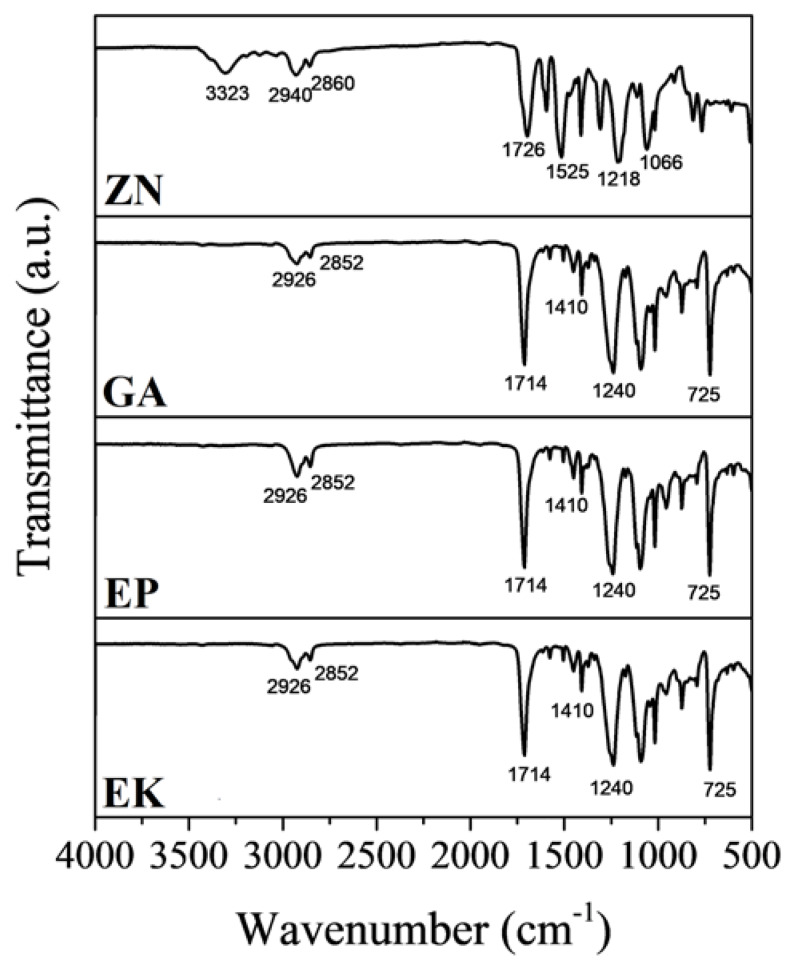
Fourier transformation infrared spectroscopy (FTIR) spectra of the thermoplastic materials.

The FTIR-ATR analyses on EK, EP and GA samples show comparable profiles with specific bands of PETG (Figure 4) [47,48,49,50,51]. In particular, the following bands are identified: the peak at 1714 cm^−1^ can be ascribed to the C=O of ester groups, and the C-H out-of-plane deformation of two carbonyl substituents on the aromatic ring at 725 cm^−1^; the two bands at 1410 and 1240 cm^−1^ related to –CH2– deformation and C(O)–O stretching of ester groups, respectively; the asymmetric and symmetric aliphatic C–H stretching vibrations are observed at 2852 and 2926 cm^−1^, due to the presence of methylene groups in the structure of PETG. 

In the ZN sample, the IR absorption bands, characteristic of the semirigid PU based-polymer, can be summarized as follows: characteristic bands of the hydroxyl and carbonyl groups in the 3323 and 1726 cm^−1^ regions, respectively; the two bands between 2940 and 2860 cm^−1^ attributed to the symmetric and nonsymmetric stretching of the C–H bond with carbonyl; stretching C = O and N–H bonds around 1700 and 1525 cm^−1^; band features of the polymerized urethanes [43].

**Figure 5:**
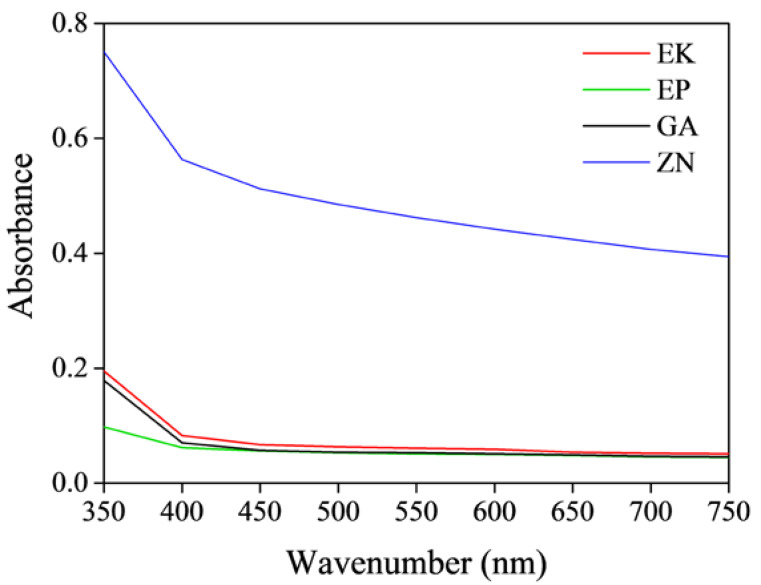
Absorbance curves of the as-received thermoplastic materials; EK sample (red line), EP sample (green line), GA sample (black line), ZN sample (blue line).

In regard to UV-VIS measures, for all the samples, similar trends are observed, showing a decrease in the absorbance values by increasing the wavenumber (Figure 5). It is possible to note that the untreated PETG-based disks show similar absorbance curves in the visible range, clearly lower than the PU-based disk. These results underline the lower transparency of the ZN disk when compared to the others, as also confirmed by visual inspections. Moreover, considering that the transparencies are related inversely to the degree of crystallinity [52], the UV-VIS results confirm the XRD measurements that reveal the highest degree of crystallinity in ZN sample.

### 3.2. Mechanical Characterization 

**Figure 6:**
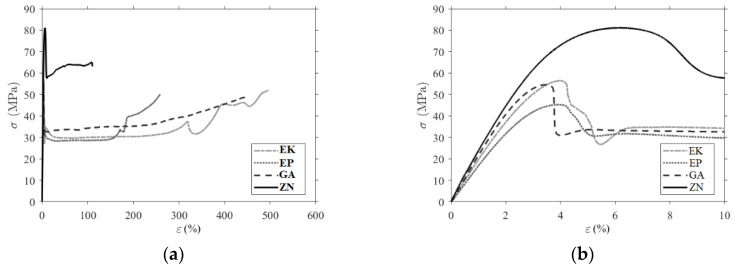
Comparison of the experimental stress–strain curves (**a**) until break; (**b**) a magnification in the elastic and post-peak phase.

The experimental curve stress—strain of the tensile tests performed are reported in Figure S3 for each material. Three tests were carried out for each material, and the figure shows the whole curve and a zoom in the initial elastic zone. A comparison of the tensile curves for the four materials is shown in Figure 6, and in Table 4, the characteristic mechanical parameters calculated according to the standard UNI EN ISO 527-1 are reported. m is the maximum stress reached during the test; εm is the strain at maximum stress; εtb is the strain at break and the maximum elongation; and E is the elastic modulus calculated as the slope of the curve between 0.05% and 0.25% of the deformation. The measured values exhibit a low standard deviation showing a good repeatability of the tests.

The characteristic mechanical parameters (the maximum stress, the strain at maximum stress, the strain at break and E the elastic modulus) calculated from the tensile test are compatible with typical values of PET and PU polymers. ZN samples exhibit the highest average values for the elastic modulus (2.49 GPa) and for the maximum stress (78 MPa) but have the lowest strain at break (95%). The three PETGs have comparable values, and the EP has the lowest modulus of elasticity (1.74 GPa) and the lowest maximum stress (45 MPa).

**Figure 7:**
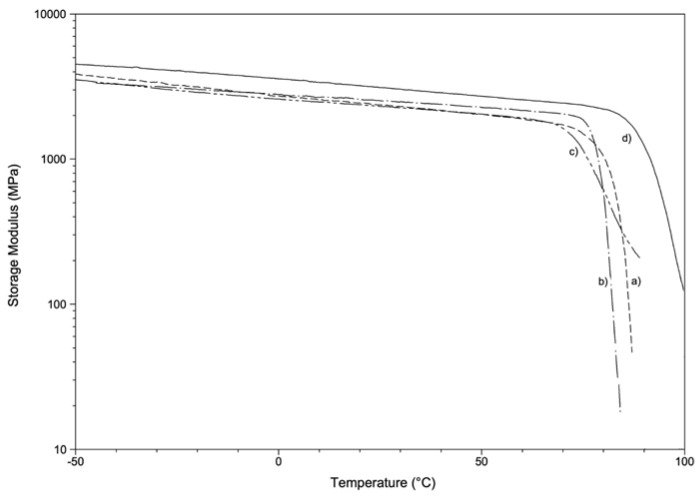
Representative storage modulus as a function of temperature: (**a**) EK, (**b**) EP, (**c**) GA, (**d**) ZN.

Representative storage modulus (E’) vs. temperature curves are reported in Figure 7. Storage modulus values at room temperature (E’RT) and glass transition temperature values (Tgonset, determined as onset at the of the E’ curve drop) are reported in Table 5.

The values of glass transition temperature detected by dynamic-mechanical analysis (Tgonset) are in good agreement with those obtained by DSC analysis, showing a slightly lower Tgonset for PETG-based samples (71.9 °C, 77.2 °C and 79.5 °C for GA, EK and EP, respectively) when compared with PU-based samples (88.1 °C for ZN). The highest Tg value showed by ZN also results in the highest value of storage modulus E’RT (2840 MPa). The dynamic modulus values for PETG-based samples are in the range 2160–2430 MPa and are compatible with typical value for rigid amorphous thermoplastic polymers. Taking into account the experimental error and the different mechanical analysis (quasi-static and oscillatory conditions), the storage modulus values are also in good agreement with the elastic modulus values reported in Table 4.

### 3.3. Colour Change Evaluations

**Figure 8:**
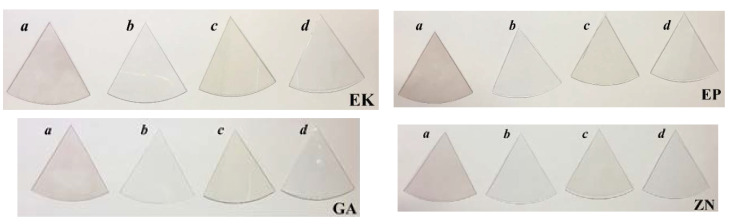
Visual inspection of the thermoplastic materials of the different brands, before and after 14 days of immersion both in artificial saliva and staining beverages. Legend: **a**: red wine; **b**: artificial saliva; **c**: coffee; **d**: nicotine solution.

The visual inspections of the disks of thermoplastic materials of the different brands before and after 14 days of immersion in the staining solutions are shown in Figure 8. For all the samples, the colour variations, as perceivable to the naked eye, were observed only when the samples are immersed in red wine or coffee, showing colours of pink and light brown, respectively.

**Figure 9:**
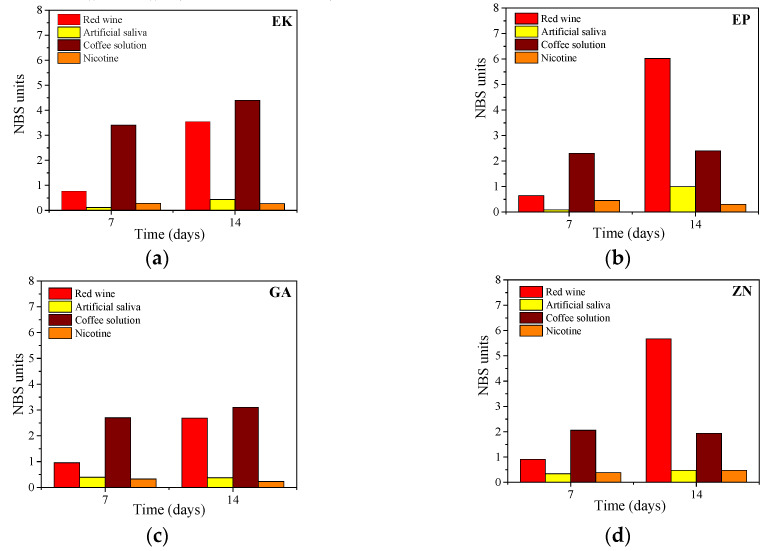
Colorimetric measurements (expressed by NBS system) of the thermoplastic disks after immersion in staining beverages for 7 and 14 days, respectively (**a**) EK, (**b**) EP, (**c**) GA, (**d**) ZN.

These results are confirmed and quantified by the colorimetric measurements, reported in Figure 9. According to the descriptions of colour changes established by the NBS system (Table 2), all the samples, maintained up to 7 days in immersion, reveal NBS values ranging from an extremely slight colour change (NBS < 0.5) to a slight change (NBS < 1.5), except for the immersion in the coffee solution. In this latter case, EP, GA and ZN samples show a perceivable colour variation (NBS < 3), while the EK sample displays marked change in the colour (NBS > 3). 

After 14 days of immersion in the nicotine solution, all the samples exhibited only slight colour changes, while when immersed in red wine, they revealed perceivable colour variations, with an increase with time, especially for EP and ZN (NBS > 3). Finally, considering the immersion in coffee solutions, marked changes in colour were observed in the EK sample, while the other disks presented only perceivable variations with NBS values ranging from 1.5 to 3.0.

**Figure 10:**
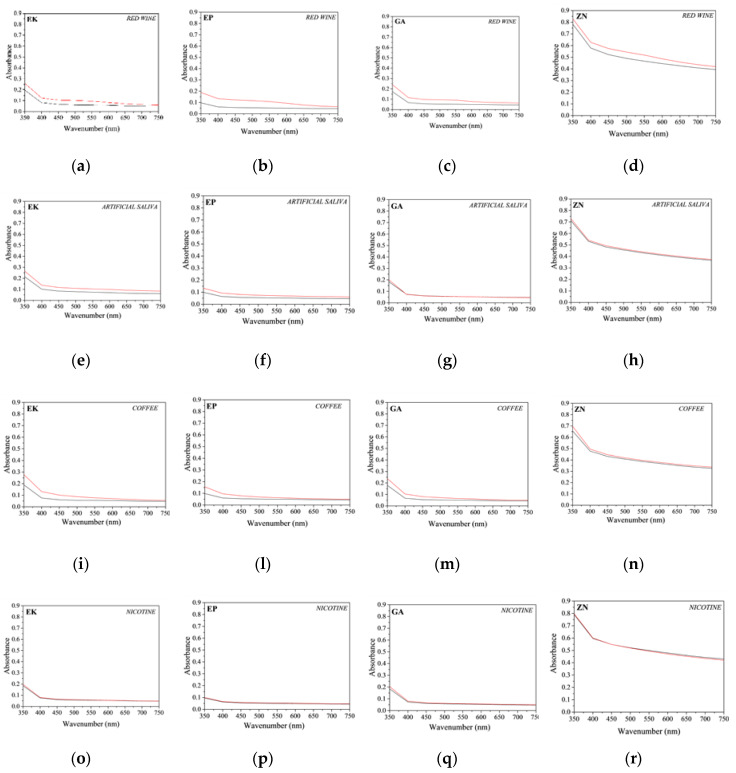
Comparison of absorbance curves of the thermoplastic disks before (black line) and after (red line) 14 days of immersion into staining agents. (**a**–**d**) red wine (EK, EP, GA and ZN samples, respectively); (**e**–**h**) artificial saliva (EK, EP, GA and ZN samples, respectively); (**i**–**n**) coffee (EK, EP, GA and ZN samples, respectively); (**o**–**r**) nicotine solution (EK, EP, GA and ZN samples, respectively).

In regard to the UV-visible measurements, the absorbance curves of the thermoplastic disks of the different brands before and after 14 days of immersion in beverage solutions are reported in Figure 10. In particular, in order to directly compare the different behaviour of brands and solutions, the graphs are characterized by the same y-scale.

For all the samples independent from the staining agent, it is possible to note a general increase in the absorbance curves, resulting in a small loss of transparency of all the samples. In particular, for the PETG-based aligners, (EK, EP and GA samples), similar behaviours were observed, and in particular, an absorbance increase of about 30% for all the analysed wavelengths was measured after immersion in red wine and coffee. On the contrary, regarding the results for ZN samples, smaller increases in the absorbance were registered, confirming the visual observations previously reported (Figure 8). 

### 3.4. Water Absorption Properties

**Figure 11:**
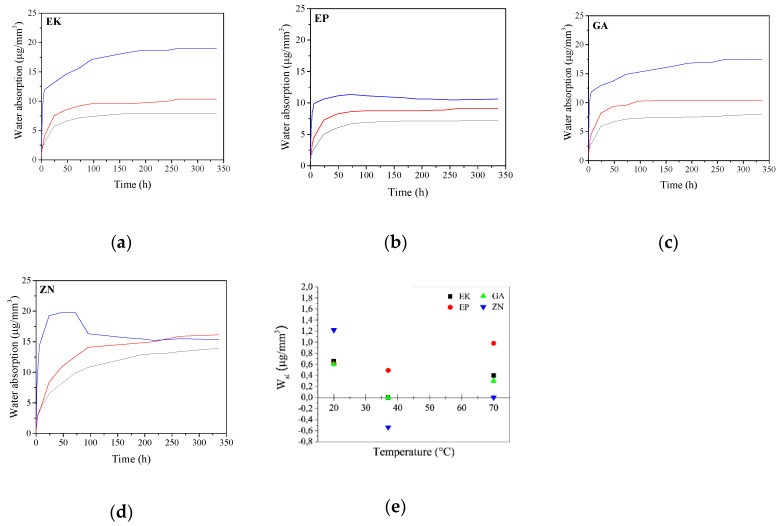
(**a**–**d**) Water absorption curves of the different samples by varying both the immersion time and temperatures. Legend: T = 20 °C (black line); T = 37 °C (red line); T = 70 °C (blue line). (**e**) Nominal water solubility of the samples (Wsl), after immersion in water at different temperatures.

In regard to the study of the influence of temperature on water absorption of the disks versus time, the curves are reported in Figure 11.

Considering the samples immersed in water at T = 20 °C, it is possible to note that all PETG-based disks (EK, EP and GA) present comparable trends reaching a hyperbolic saturation value of water absorption of about 8 μg/mm^3^. On the contrary, the curve of the PU polymer (ZN sample) does not reach a plateau in the considered time interval showing, in addition, the highest water absorption value among all the samples (up to 14 μg/mm^3^). The obtained results can be related to the different chemical composition of the two groups of the considered samples, causing different behaviour in the penetration of water molecules into the material structure. Considering that the penetration of water in the polymeric disk is responsible for swelling and mechanical degradation phenomena, which in turn leads to physicochemical changes in the material itself [19,20,21], these results could be very important because the degradation will be more evident when increasing the water absorption ability of the material itself. 

Considering the immersion in water at 37 °C, all the curves show similar trends in respect to those obtained at 20 °C. Nevertheless, it is possible to note that the samples generally reach higher saturation values ranging from about 10 to 16 μg/mm^3^ for PETG- and PU-based polymers, respectively, due to the role of temperature on the higher diffusion of water molecules inside the materials. After the immersion in water at 70 °C, PETG-based disks reveal an increase in the water absorption, particularly evident for the EK and GA samples, which reach final values of about 17 μg/mm^3^. On the contrary, ZN presents an initial increase in the amount of water absorbed, reaching the maximum value within the first 75 h of immersion. Subsequently, the curve shows a decrease in the absorbed water of up to about 15 μg/mm^3^. This decrease in weight could be related to a loss of polymer molecules with time; if this hypothesis was true, the effective amount of water absorbed should be about 20 μg/mm^3^, corresponding to the hyperbolic saturation behaviour. 

In parallel, in regard to the Wsl results, the obtained data are reported in Figure 11e. We can observe that at 20 °C, all the samples reveal a positive Wsl that is a variation in weight after the immersion, which is likely due to absorbance of water rather than real solubilisation of the material. In particular, the ZN sample shows the highest values (1,22 μg/mm^3^) followed by EK, EP and GA (with 0.66, 0.60 and 0.59 μg/mm^3^, respectively). When increasing the temperature up to 37 °C, that is, the temperature relivable in the oral cavity, the EP sample reveals less variation in weight (observed as 20 °C (Wsl of 0.5 μg/mm^3^)), while the EK and GA samples present values of next to 0 μg/mm^3^, underlining a material loss, particularly evident for the ZN sample showing negative values as well. These results can be justified considering that the remaining water was probably trapped inside the samples after immersion, entering the polymeric lattice in an irreversible way and causing modification of the properties of the material itself. Finally, after the immersion at 70 °C, the Wsl values increase for all the samples, confirming that they are strictly related to the balance between the real solubility of the material and its ability to absorb water during the test. 

In conclusion, the results obtained from the water absorption test underline that the temperature, also at relatively low values (37 °C), is a critical factor both on the diffusion of water inside the material and in the thermal degradation of the polymer itself, weakening and breaking the bonds between the chains and causing possible degradation with time in terms of mechanical properties. 

From the visual observations, after the immersion at 20 °C and 37 °C, all the samples remain unaltered. On the contrary, when immersed at 70 °C, they present clear changes visible to naked eye after just 1 h from the beginning of the immersion tests (Figures S4 and S5). In particular, EK sample tends to bend and show a visible change in colour, turning from transparent to white in 6 days (Figure S4b). Additionally, after 6 days of immersion, the presence of bubbles and microcracks on the disk surface is evident, probably due to the increase in the amount of water entering the sample, as discussed before. The same results are obtained from the EP sample, showing a loss of transparency together with the formation of superficial bubbles that after 6 days of immersion at 70 °C (Figure S4c,d). The GA sample shows a clear mechanical deformation after just 15 min from the beginning of the test (Figure S5a), reaching a colour variation from transparent to white combined with the formation of superficial bubbles after 6 days of immersion (Figure S5b). From the visual inspection of the ZN disk, a higher stiffness in terms of mechanical deformation is evident, showing only slight curvatures after 15 min of immersion, which remain unaltered until the end of the test (Figure S5d). Moreover, ZN samples do not reveal chromatic variations in colour, indicating a lower degradation of this polymer compared to the other brands when exposed to high temperatures. 

**Figure 12:**
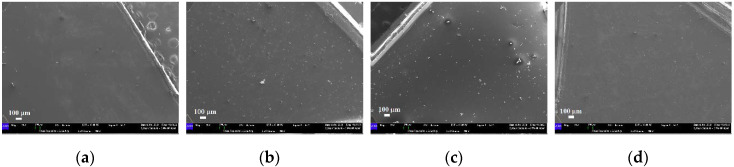

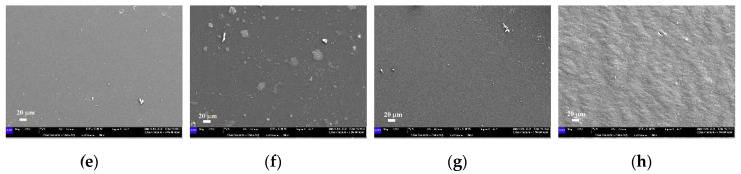
Scanning electron microscopy (SEM) micrographs of the samples, as received: (**a**–**e**) EK disk (magnifications 80× and 500×, respectively); (**b**–**f**) EP disk (magnifications 80× and 500×, respectively); (**c**–**g**) GA disk (magnifications 80× and 500×, respectively); (**d**–**h**) ZN disk (magnifications 80× and 500×, respectively).

SEM images related to the samples disks as received, at two different magnifications, are shown in Figure 12. At a higher magnification, the surface of the EK sample (Figure 12a–e) appears rather smooth with small and sporadic superficial impurities or irregularities and ununiformly distributed. The EP and GA samples are characterized by a smooth surface too, but differently from what was observed in the EK sample, they present a larger number of impurities or irregularities on their surfaces (Figure 12b–f). However, in the case of the EP sample, such irregularities are in relief and characterized by a circular morphology, particularly evident at high magnification, with typical dimensions ranging from 5 to 50 μm. Finally, in regard to the polyurethane-based disk, the ZN sample surface presents rare impurities, but differently from what was observed in all the PETG-based disks, it appears wrinkled (particularly visible at high magnification) and is characterized by some small cavities (Figure 12d–h).

**Figure 13:**
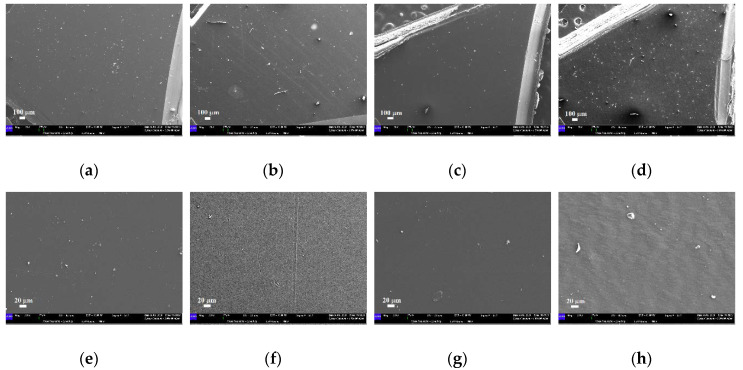
SEM micrographs of the samples immersed in water at 37 °C: (**a**–**e**) EK disk (magnifications 80× and 500×, respectively); (**b**–**f**) EP disk (magnifications 80× and 500×, respectively); (**c**–**g**) GA disk (magnifications 80× and 500×, respectively); (**d**–**h**) ZN disk (magnifications 80× and 500×, respectively).

When immersed in water at different temperatures, all the samples show variations in the surface features, already evident at 37 °C. Specifically, after the immersion for 14 days at 37 °C, a general increase in the amount of impurities and raised irregularities is observed on the PETG-based samples (Figure 13a–c). Moreover, several ripples are recognizable too in the EP sample (Figure 13b–f) that are not visible in the as-received disk. Furthermore, the ZN sample reveals an increase in the irregularities present on its surface, together with a reduction in the wrinkled morphology, Figure 13d–h. 

**Figure 14:**
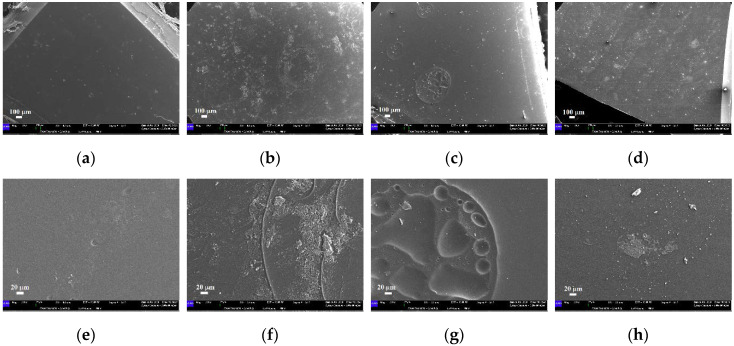
SEM micrographs of the samples immersed in water at 70 °C: (**a**–**e**) EK disk (magnifications 80× and 500×, respectively); (**b**–**f**) EP disk (magnifications 80× and 500×, respectively); (**c**–**g**) GA disk (magnifications 80× and 500×, respectively); (**d**–**h**) ZN disk (magnifications 80× and 500×, respectively).

Considering the images of the samples immersed in water at 70 °C, EK and GA disks remain similar, presenting large bubbles together with the formation of localized cavities, (Figure 14a–e,c–g, respectively). The EP sample shows noticeable superficial changes with the formation of several microbubbles, cavities and a high number of impurities, homogeneously distributed and characterized by an irregular morphology (Figure 14b–f). Finally, large bends, parallel placed and homogenously distributed, appear on the surface of the ZN sample; on these bends all the impurities and irregularities seem to be localized (Figure 14d). At higher magnification, the surface after immersion at 70 °C always appears smooth, (Figure 14h), when compared to those observed at lower temperatures, which is in agreement with the ability of water to be absorbed inside the polymeric structure causing the swelling phenomena. Both the results on the PETG-based disks and on the PU-based one can be related to the polymer degradation caused by the simultaneous action of temperature, as also reported in the literature [22].

From the comparative analyses carried out in this work, the PETG disks present small differences when exposed to the same external conditions, despite the differences in cost they have on the market. On the contrary, the polyurethane disk, regardless of its advantageous mechanical and thermal resistance, showed a limited transparency, and when immersed in water, a great ability to absorb water and to release small quantities of product by increasing temperature. 

In conclusion, this paper represents a detailed guide which can be used, in addition to the manufacturers’ technical sheets, for a more appropriate choice of materials to use to realise clear orthodontic aligners, taking into account all the aspects and features from the mechanical to the physicochemical point of view. Furthermore, this paper can be the basis for further in-depth studies on these materials or for innovative materials to be used in the orthodontic field.

## 4. Conclusions

This paper aims to provide an overview on the physicochemical and mechanical characterization of thermoplastic disks used to realise orthodontic invisible aligners. Disks from different brands are considered based on the most used polymeric materials, PETG (EK, EP and GA brands) and PU (ZN brand). The analyses reveal that the PU disk presents the highest crystallinity as well as the highest mechanical strength and glass transition temperature among all the samples. Instead, all the PETG-based disks are characterized by similar crystallinity, mechanical resistance and thermal stability. From UV-VIS measurements, the PETG disks show similar absorbance curves, and they present lower values in comparison with the PU disk, underlining a better transparency, as also confirmed by visual inspections and crystallinity values. The immersions in staining beverages reveal that red wine and coffee solutions give the highest colour variations, while nicotine and artificial saliva show negligible colour variations. Overall, the GA disk presents colour alterations lower than the other disks, as confirmed by the UV-VIS analyses performed after staining beverages immersions.

In regard to the water absorption ability versus time, all the PETG disks present a trend of hyperbolic saturation that increases with temperature and with an amount of absorbed water at 37 °C of about 8 µg/mm^3^. Specifically, the EP sample reveals the lowest ability to absorb water with increasing temperature. On the contrary, the PU disk presents not only higher amounts of absorbed water (15 µg/mm^3^ at 37 °C), but also a different trend in the saturation behaviour at 70 °C, revealing a possible small dissolution in the liquid phase. In this respect, as shown from the nominal water solubilities measurements, the EP disk presents the lowest dissolution aptitude. This result could provide important information because the penetration of water causes swelling and mechanical degradation phenomena, so a high-water absorption ability corresponds to a higher aptitude to degradation of the material itself. SEM observations confirm the swelling phenomena, more evident in the surface of the PU sample that changes from a wrinkled to a rather smooth appearance with increasing temperature. 

## Figures and Tables

**Table 1:** Main characteristics of the thermoplastic materials used in the present study.

Sample Name	Brand (Manufacturer) *	Chemical Composition
EK	Erkodur (Erkodent Erich Kopp GmbH, Pfalzgrafenweiler, Deutschland)	polyethylene terephthalate glycol (PETG)
EP	Essix Plastic (Dentsply Sirona, York, PA USA)	polyethylene terephthalate (PET)
GA	Ghost Aligner (BART MEDICAL S.r.l., Mezzano, Italy)	polyethylene terephthalate (PET)
ZN	Zendura (Zendura, Bay Materials LLC, Fremont, CA, USA)	polyurethane (PU)

* The manufacture’s specification, when available, were reported in [23,24,25].

**Table 2:** Description of colour changes from the National bureau of standard units.

**National Bureau of Standards Units**	**Descriptions of Colour Changes**	NBS = 0.92 × ΔE*
0.0–0.5	Trace: extremely slight change	
0.5–1.5	Slight: slight change	
1.5–3.0	Noticeable: perceivable	
3.0–6.0	Appreciable: marked change	
6.0–12.0	Much: extremely marked change	
12.0 or more	Very much: change to another colour	

**Table 3:** Full width at half maximum values (FWHM) of the as-received thermoplastic materials of the different brands.

Sample	EK	EP	GA	ZN
FWHM (° 2θ)	9.643	8.148	9.695	6.313

**Table 4:** Mechanical parameters calculated according to the standard UNI EN ISO 527-1: σ_m_ is the maximum stress, εm is the strain at maximum stress, εtb is the strain at break and E is the elastic modulus. μ is the mean value, σ is the standard deviation and C_v_ is the coefficient of variation.

Sample	σ_m_ (MPa)			ε_m_ (%)			ε_tb_ (%)			E (MPa)		
	μ	σ	c_v_	μ	σ	c_v_	μ	σ	c_v_	μ	σ	c_v_
EK	55.93	0.42	0.8%	3.99%	0.13%	3.3%	507%	59%	11.6%	1933.03	130	6.7%
EP	45.37	0.54	1.2%	3.91%	0.02%	0.4%	261%	5%	1.8%	1742.03	46	2.6%
GA	53.65	0.85	1.6%	3.32%	0.15%	4.5%	396%	58%	14.7%	2102.83	24	1.1%
ZN	78.20	5.25	6.7%	6.22%	0.05%	0.8%	95%	30%	31.7%	2489.43	74	3.0%

**Table 5:** Dynamic-mechanical analysis: Storage modulus at room temperature (E’RT) and glass transition temperature (Tgonset, determined as onset at the of the E’ curve drop).

Sample	EK	EP	GA	ZN
E’_RT_ (MPa)	2430	2160	2280	2840
Tg_onset_ (°C)	77.2	79.5	71.9	88.1

## Supplementary Materials

The following are available online at https://www.mdpi.com/1996-1944/13/10/2386/s1, Figure S1: The as-received thermoplastic materials properly cut into six equal segments, Figure S2: DSC results for (a) EK, (b) EP, (c) GA, (d) ZN thermoplastic materials, respectively, Figure S3: Experimental stress-strain curves: (a-b) EK, (c-d) EP, (e-f) GA, (g-h) ZN, Figure S4: Visual observations of EK and EP samples after immersion in water at 70°C: (a-c) comparison between the as-received sample (left) and the disk immersed for 1 hour (right); (b-d) samples after 6 days of immersion, Figure S5: Visual observations of GA and ZN samples after immersion in water at 70°C: (a-c) comparison between the as-received sample (left) and the disk immersed for 15 minutes (right), respectively; (b-d) samples after 6 days of immersion.
