# Supplementary material for: Thermoplastic Disks Used for Commercial Orthodontic Aligners: Complete Physicochemical and Mechanical Characterization
Source: Materials (Basel). 2020 May 22;13(10):2386. doi: 10.3390/ma13102386 (PMC7287673; doi:10.3390/ma13102386)
Supplement: Supplementary file 1 [file materials-13-02386-s001.pdf]

materials

Supplementary Materials: Thermoplastic Disks Used for Commercial Orthodontic Aligners: Complete Physicochemical and Mechanical Characterization

Valeria Daniele [1], Ludovico Macera [1,*], Giuliana Taglieri [1], Alessandra Di Giambattista [1], Giuseppe Spagnoli [1], Alessandra Massaria [2], Massimo Messori [3], Enrico Quagliarini [4], Gianluca Chiappini [5], Vincenzo Campanella [6], Stefano Mummolo [2], Enrico Marchetti [2], Giuseppe Marzo [2] and Vincenzo Quinzi [2]

[1] Department of Industrial and Information Engineering and Economics, University of L'Aquila, Piazzale Pontieri 1, Monteluco di Roio, 67100 L'Aquila, Italy; valeria.daniele@univaq.it (V.D.); giuliana.taglieri@univaq.it (G.T.); digiambaale@hotmail.it (A.D.G.); giuseppe.spagnoli@univaq.it (G.S.);
[2] Department of Life, Health & Environmental Sciences, Postgraduate School of Orthodontics, University of L'Aquila, P.le Salvatore Tommasi 1, Ed. Delta 6, 67100 Coppito L'Aquila, Italy; massariaalessandra@gmail.com (A.M.); stefano.mummolo@cc.univaq.it (S.M.); enrico.marchetti@cc.univaq.it (E.M.); giuseppe.marzo@univaq.it (G.M.); vincenzo.quinzi@univaq.it (V.Q.)
[3] Department of Engineering 'Enzo Ferrari', University of Modena and Reggio Emilia, Via P. Vivarelli 10, 41125 Modena, Italy; mmessori@unimore.it
[4] Department of Construction, Civil Engineering and Architecture, Polytechnic University of Marche, 60121 Ancona, Italy; e.quagliarini@staff.univpm.it
[5] Department of Industrial Engineering and Mathematical Sciences, Polytechnic University of Marche, via Brecce Bianche snc, 60131 Ancona, Italy; g.chiappini@univpm.it
[6] Department of Clinical Science and Translational Medicine, University of Rome "Tor Vergata", Via Montpellier 1, 00133 Roma, Italy; vincenzo.campanella@uniroma2.it
* Correspondence: ludovico.macera@graduate.univaq.it

Supplementary Materials

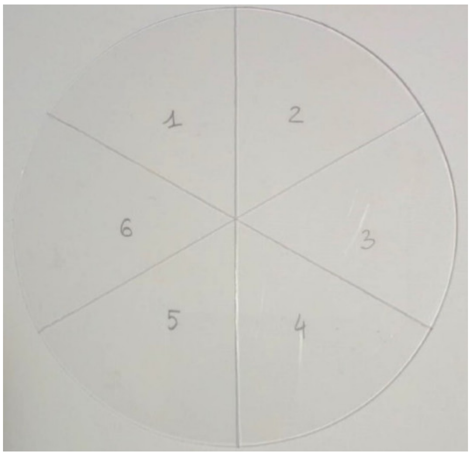

Figure S1. The as-received thermoplastic materials properly cut into six equal segments.

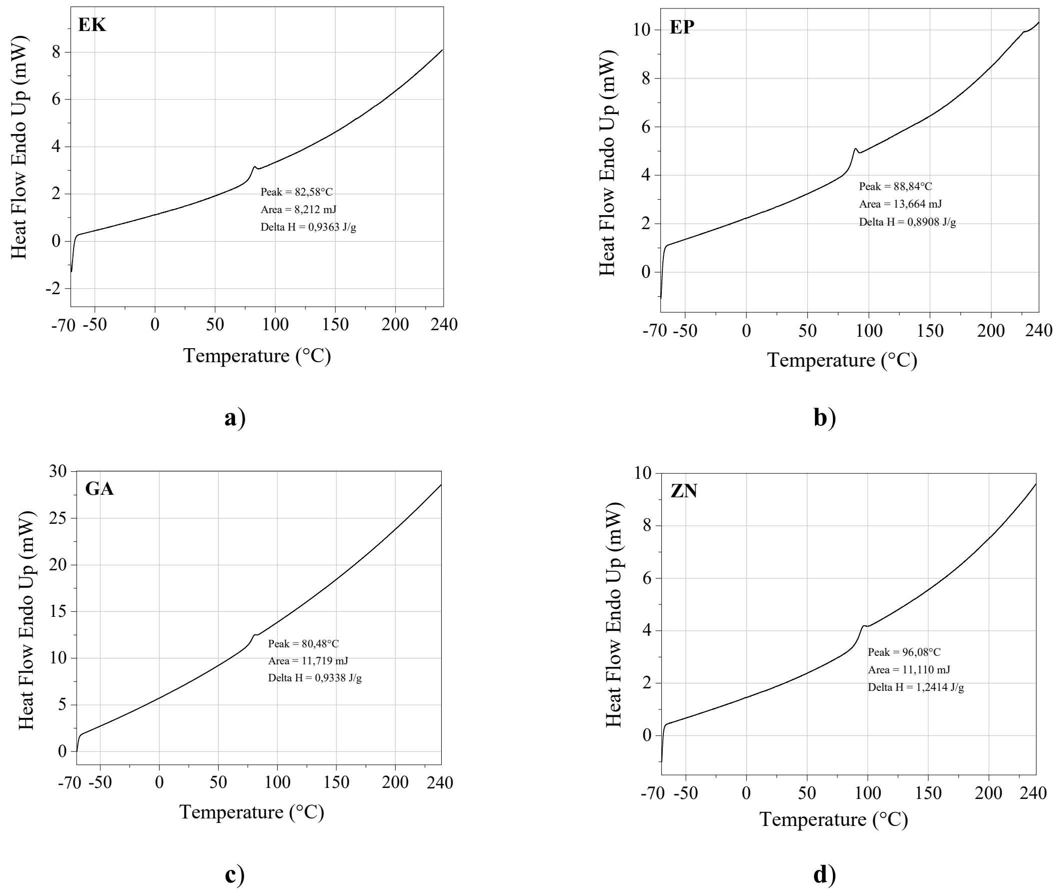

Figure S2. DSC results for: **a**) EK, **b**) EP, **c**) GA, **d**) ZN thermoplastic materials, respectively.

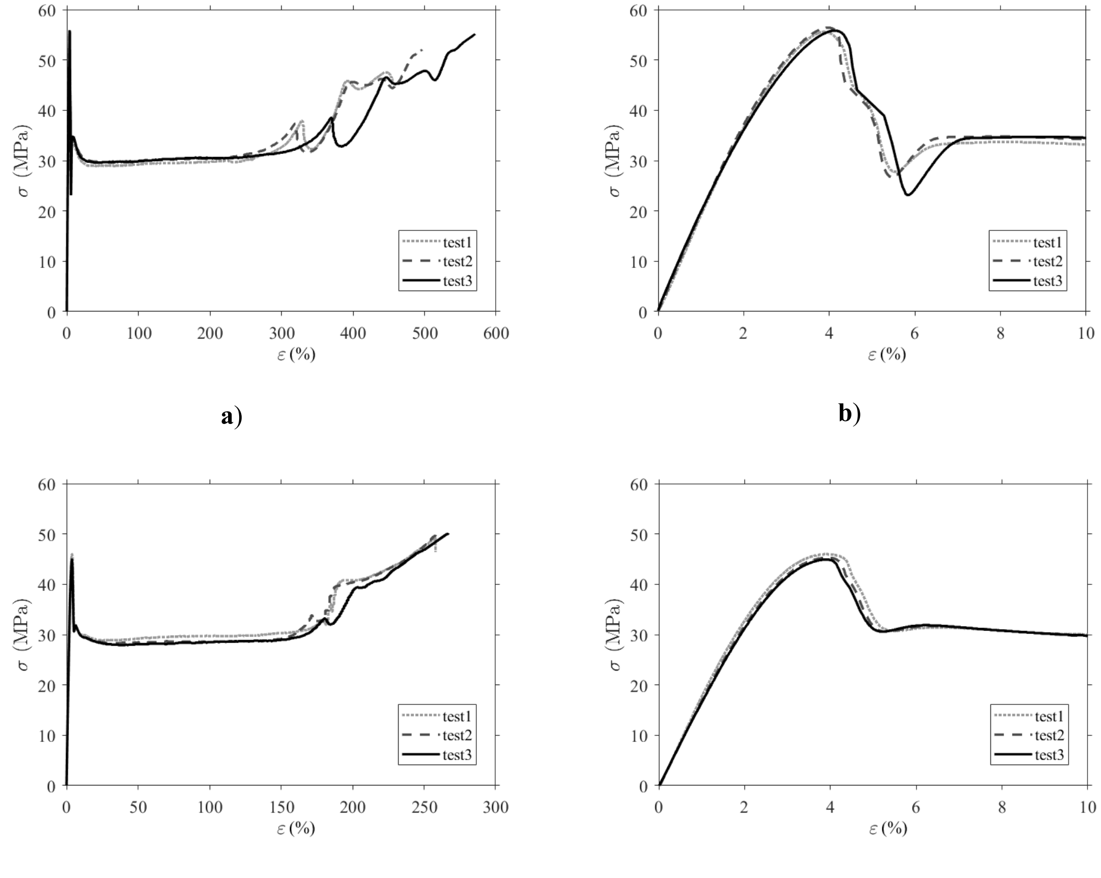

c)

d)

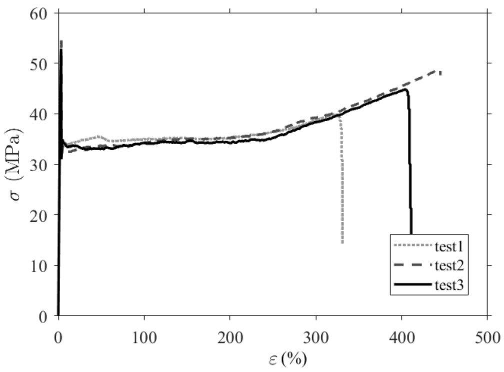

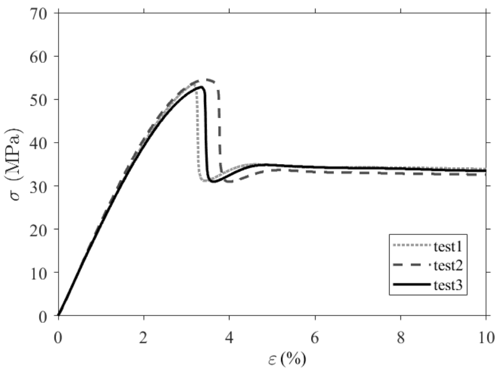

e)

f)

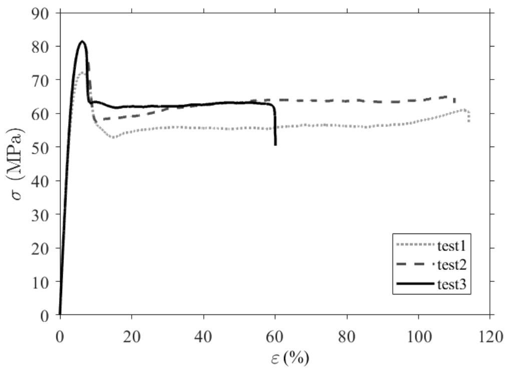

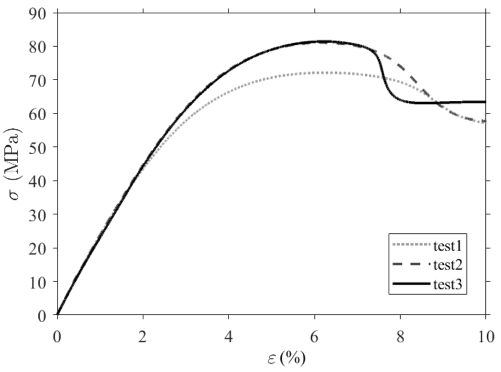

g)

h)

Figure S3. Experimental stress-strain curves: **a**-**b**) EK; **c**-**d**) EP; **e**-**f**) GA; **g**-**h**) ZN.

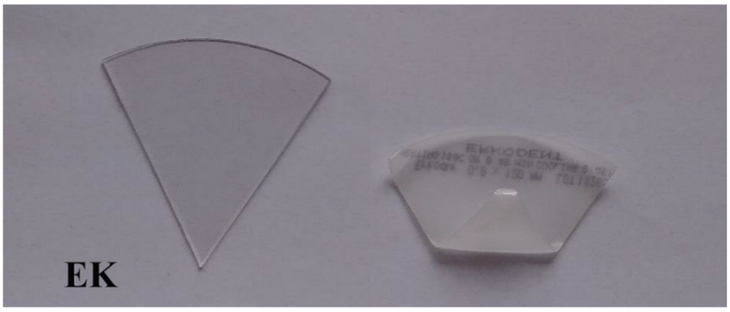

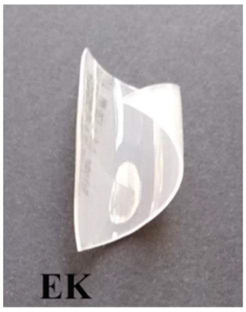

a)

b)

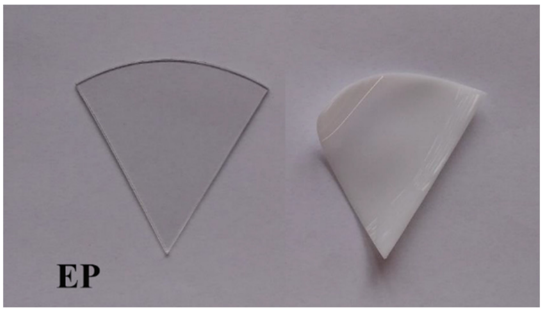

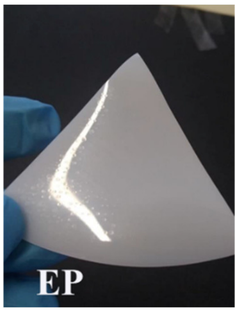

c)

d)

Figure S4. Visual observations of EK and EP samples after immersion in water at 70 °C: **a**-**c**) comparison between the as-received sample (left) and the disk immersed for 1 h (right); **b**-**d**) samples after 6 days of immersion.

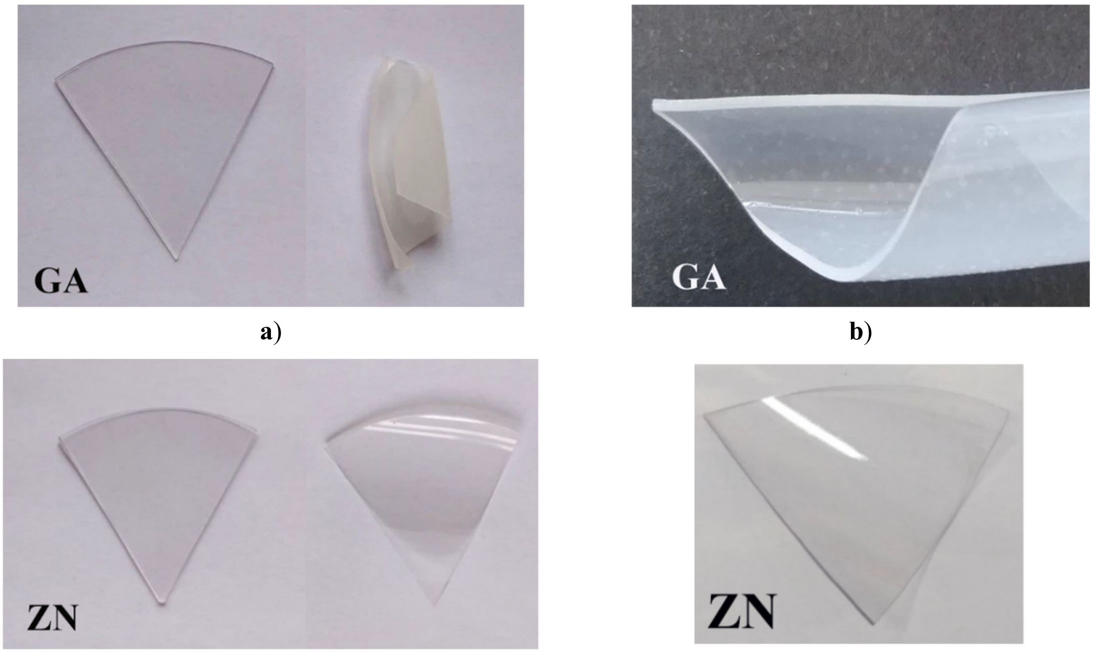

a) **b**)

c) **d**)

Figure S5. Visual observations of GA and ZN samples after immersion in water at 70 °C: **a**-**c**) comparison between the as-received sample (left) and the disk immersed for 15 min (right), respectively; **b**-**d**) samples after 6 days of immersion.

© 2020 by the authors. Submitted for possible open access publication under the terms and conditions of the Creative Commons Attribution (CC BY) license (http://creativecommons.org/licenses/by/4.0/).
